# Prevalence and Correlates of Polypharmacy, and Drug Utilization Pattern in a Semi-urban Population: Results from the Pars Cohort Study

**DOI:** 10.34172/aim.2023.24

**Published:** 2023-03-01

**Authors:** Mohammad Reza Akbari, Alireza Kamalipour, Mahsa Pakroo, Bita Mesgarpour, Hossein Poustchi, Zahra Mohammadi, Abdullah Gandomkar, Hossein Molavi Vardanjani

**Affiliations:** ^1^MD-MPH Department, School of Medicine, Student Research Committee, Shiraz University of Medical Sciences, Shiraz, Iran; ^2^Hamilton Glaucoma Center, Shiley Eye Institute, Viterbi Family Department of Ophthalmology, University of California, San Diego, California, United States; ^3^Student Research Committee, Shiraz University of Medical Sciences, Shiraz, Iran; ^4^National Institute for Medical Research Development (NIMAD), Tehran, Iran; ^5^Digestive Disease Research Center, Digestive Research Institute, Shariati Hospital, Tehran University of medical Science, Tehran, Iran; ^6^Non-communicable Disease Research Center, Shiraz University of Medical Sciences, Shiraz, Iran; ^7^MD-MPH Department, School of Medicine, Research Center for Traditional Medicine and History of Medicine, Shiraz University of Medical Sciences, Shiraz, Iran

**Keywords:** Irrational drug use, Medication profile, Polypharmacy, Socioeconomic status

## Abstract

**Background:**

Although polypharmacy is considered a major predictor of irrational use of drugs, little is known about polypharmacy in developing regions. We aimed to indicate the prevalence and correlates of polypharmacy and to determine the medication profile at the population level in southern Iran.

**Methods:**

In this cross-sectional study, we analyzed data from participants of the Pars Cohort Study (PCS) (aged above 40 years, N=9269). Polypharmacy was defined as using five or more medications concurrently. A Poisson multivariable model was applied to estimate the adjusted prevalence ratios (APRs) of various risk factors. The Anatomical Therapeutic Chemical (ATC) classification system was used for classifying medications.

**Results:**

Prevalence of polypharmacy was 10.4%, (95% CI: 9.75; 11.08) and it was higher among females (15.0%), older adults (age≥65 years) (16.0%), and individuals with more than two chronic conditions (31%). Being female, educated, married, and not having a low socio-economic class were independently associated with a higher likelihood of polypharmacy. The most prevalent medications among female participants were sex hormones and modulators of the genital system (58.4%), drugs for acid-related disorders (14.6%), and anti-anemic preparations (13.6%,). On the other hand, males were using acid-related disorders (14.6%), anti-inflammatory and anti-rheumatic products (7.8%), and beta-blocking agents (6.3%).

**Conclusion:**

The prevalence of polypharmacy in our sample was relatively low, especially among males. Cardiovascular drugs, acid suppressants, hormonal contraceptives, and anti-anemic preparations are drug classes with the highest contribution to polypharmacy.

## Introduction

 Inappropriate consumption of drugs, including irrational medication use and underuse, is a serious health problem. The World Health Organization (WHO) has estimated that over half of all prescribed, sold, or dispensed drugs are inappropriate, and only half of all patients take their medicines as prescribed or dispensed.^1^ This is harmful to both patients and communities by compromising patients’ safety and increasing healthcare costs.^2^

 Polypharmacy, defined as the concurrent use of multiple medications by an individual, is the major correlate of taking a potentially inappropriate prescribing medicine.^3,4^ It increases the probability of adverse drug reactions, drug-drug interactions, and medication non-adherence.^5^ However, polypharmacy can be essential and its absence should not be considered as evidence of rational use of medicine.

 The prevalence of polypharmacy in adults ranges from 10 to 90%^6^ and is associated independently with biological factors, such as age and multi-morbidity; socio-demographic factors, such as education and gender; health-related behavior factors, such as smoking; and also several healthcare system-related factors, such as the quality of medical record-keeping.^7-9^ Based on this multifactorial nature, the epidemiology of polypharmacy varies across countries and even across different regions inside each country.^5^

 In low- and middle-income countries, monitoring of drug utilization is not adequately integrated into the healthcare system. Therefore, these populations may be exposed to a greater risk of irrational use of medicine.^10^ Additionally, the economic burden of inappropriate use of medication may be larger for low- and middle-income countries, because in such regions, 25%–70% of healthcare expenditure is spent on medicines, compared to less than 10% in most high-income countries.^11^ However, a few population-based, high-quality studies have investigated polypharmacy and irrational use of medicine in these regions.^12^

 Limited research explores polypharmacy in Iran. The reported prevalence of polypharmacy in community-dwelling Iranian older adults ranges from 39.6% to 56.6%.^13^ A recent report showed a very low prevalence of polypharmacy (0.24%) among 20–65 years old adults in the Khuzestan province in southwestern Iran.^14^ In this study, we analyzed baseline data from the Pars Cohort Study (PCS) to investigate the prevalence and correlates of polypharmacy and to determine the medication profile among the general population and among those with polypharmacy.

## Materials and Methods

###  Study Design, Setting, Participants

 We used baseline data from the PCS in this large-scale population-based cross-sectional study. The PCS is an ongoing cohort study investigating the major determinants of non-communicable diseases in an adult population (older than 40 years) living in a less-developed region located in southwestern Iran. Inclusion criteria were willingness to participate in the study, written informed consent, and not being a temporary resident. With a response rate of 95%, a total of 9269 participants enrolled in the PCS. More details of the design and procedures of the PCS have been described elsewhere.^15^

###  Variables and Data Collection

 The PCS Baseline measurements, which took place from 2012 to 2014, included a face-to-face interview, a physical examination, anthropometric measurements, and blood and nail sampling.^15^ Data collection was done by local physicians and trained nurses using standardized questionnaires and calibrated equipment.

 Every participant was asked to bring all the medications, including over-the-counter (OTC) and complementary medicines that he or she is regularly taking at the time of the interview. A trained nurse checked the drugs in the bag with the participants and recorded drug data. Polypharmacy was defined as using at least five different medications concurrently.

###  Possible Correlates

 To identify polypharmacy correlates, a list of variables was selected by applying a conceptual model designed based on the most available evidence and discussions made in the research team. For the conceptual modelling, factors that directly or indirectly, leading to or preventing polypharmacy were considered as its correlates. This is a simplified model truly, and we know that bidirectional cause-effect relationships and also other complex relationships are possible between polypharmacy and some of these potential correlates. As a large number of variables related to socio-demographic characteristics, health-related behaviors, medical history, anthropometric indicators, and biological indices were measured in the baseline evaluations of the PCS, we had the opportunity to analyze the association of polypharmacy with most of the potential correlates included in our conceptual model.

 Variables included in the analyses were age, gender, marital status, physical activity, body mass index (BMI), waist-to-height ratio, waist-to-hip ratio, socio-economic status (SES), ethnicity, level of education, cigarette smoking, tobacco smoking, opium consumption, alcohol consumption, multi-morbidity, fasting low-density lipoprotein (LDL), triglyceride (TG), and high-density lipoprotein (HDL).

 SES was determined based on a latent variable estimated by analyzing the participants’ assets, applying multiple correspondence analysis. Participants were ranked and categorized into quartiles of this latent factor (low, low-middle, middle-high, and high). Physical activity was measured by estimating the metabolic equivalent of task (METs) using data collected based on the International Physical Activity Questionnaire. Participants were ranked and categorized into three tertiles of METs. Participants’ marital status (alone [including divorced, widowed, or single], and married); educational level (illiterate and literate); and ethnicity (Persian and non-Persian) were dichotomized. BMI was calculated and categorized based on the WHO recommendations.^16^ Ever use of tobacco, cigarette, and opium was defined as at least six months of consecutive consumption throughout the participants’ life, and current use was defined as weekly consumption in the last six months. The waist-to-height ratio, as a marker of central obesity, was defined as normal for values less than 0.55.^17^ Waist-to-hip ratios of more than 0.9 in females and more than 0.8 in males were categorized as at-risk.^18^ HDL was categorized into high risk (< 40 mg/dL) and low risk (≥ 40 mg/dL). Participants were categorized into four groups based on their TG as normal (< 150 mg/dL), mildly elevated (150–199 mg/dL), moderately elevated (199–499 mg/dL), and higher than 500 mg/dL. Values of < 100 mg/dL, between 100 and 130 mg/dL, and more than 130 mg/dL were considered to classify the participants’ LDL. Measurement details are available in the PCS cohort profile.^15^

 Multi-morbidity was addressed based on the number of chronic conditions that a participant currently has. The presence of these chronic disorders is mainly determined by the participant self-reporting of conditions that have already been diagnosed by their physicians. These conditions included diabetes mellitus, stroke, cancer, chronic lung disease, atherosclerotic cardiovascular disease, hypertension, chronic liver disease, end-stage renal failure, major depressive disorder, and general anxiety disorder. We also included severe gastroesophageal reflux disease, irritable bowel syndrome, and functional constipation based on clinical criteria presented elsewhere.^19-21^ Participants were classified into three categories, without chronic disease, with one or two chronic diseases, and with more than two chronic conditions (multi-morbidity).

###  Medication Classes

 All drugs were classified using the first and second levels of the Anatomical Therapeutic Chemical (ATC) classification system.^22^ In this system, drugs are classified into groups at five different levels, and a unique code is assigned to every active substance of drugs. The code given to metformin, for example, is A10BA02, which means its first level class is alimentary tract and metabolism (A) and its second level class is drugs used in diabetes (A10). For better understanding of the popularity of sex hormones and modulators of the genital system (G03), we also determined the third level class for these drugs. Unlike OTC medications, complementary medicines are excluded from this part of our study because it is not possible to classify them according to the ATC classification system. We also calculated the share of each drug class in all the medications consumed by the study participants. This share was calculated among males, females, older adults, and also those who were on polypharmacy.

###  Statistical Analysis

 Data were cleaned using appropriate statistical techniques. Mean ± standard deviation (SD) and frequency were used to describe the data. The prevalence of polypharmacy and its 95% confidence interval (CI) were estimated assuming the Poisson distribution. The age-standardized prevalence rate (ASR) of polypharmacy was estimated considering the standard world population (WHO 2000–2025). Univariate analyses were done using chi-square test. Poisson multivariable modeling (with robust standard errors) was applied to investigate the independent association of different independent variables with polypharmacy. To do this, the variable selection was made based on a univariate *P* value of less than 0.3. The final multivariable model was fitted by applying a backward elimination technique. Adjusted prevalence ratios (APRs) and their 95% CI were estimated. A two-sided *P *value of less than 0.05 was defined as a statistically significant level. Participants with missing data of a variable were excluded from all analyses involving that variable.

 Sensitivity analyses were done. To investigate the effect of different drug classes on the association of polypharmacy with its potential determinants, we defined several variables as polypharmacy, excluding one or more drug classes. As an example, exclusion of cardiovascular drug class from the definition of polypharmacy provides a possibility for us to investigate if the significant correlation of SES with polypharmacy is mainly due to the correlation of SES with the pattern of cardiovascular drugs or not. Data analysis was done using Stata software (Release 11, College Station, TX: Stata Corp LLC).

## Results

###  Characteristics of the Population Study and Prevalence of Polypharmacy

 A total of 9269 participants with a mean age of 53.2 ± 9.7 enrolled in the study. The majority were female (53.8%) and Persian (56.3%). The prevalence of multi-morbidity was 19% (11% in males and 25% in females).

 Sixty-four percent (n = 5926) of participants had taken at least one medication; 10.40% (n = 964; 95% CI: 9.75% to 11.08%) of participants were on polypharmacy. The overall ASR of polypharmacy was 11.2% (95% CI: 10.4% to 11.2%). ASR was 5.8% (95% CI: 5.0% to 6.6%) for males and 15.7% (95% CI: 14.6% to 16.8%) for females. There was a significant difference between the prevalence of polypharmacy in males (5.0%, 95% CI: 4.1% to 5.5%) and females (15.0%, 95% CI: 14.2% to 16.3). The prevalence of polypharmacy among the study subgroups is presented in Table 1.

**Table 1:** Characteristics of the Study Participants and the Prevalence of Polypharmacy among Different Subpopulations

**Variable**	**Both Genders**		**Males, n=4277 (46%)**		**Females, n=4991 (54%)**	
	**No. (%)**	**Polypharmacy n** **(prevalance %, 95% CI)**	**No. (%)**	**Polypharmacy n prevalance (%)**	**No. (%)**	**Polypharmacy n prevalance (%)**
Age group (y)						
40–49	4209(45)	312(7.4,6.6–8.3)	1914(45)	50(3)	2295(46)	262(11)
50–64	3878(42)	463(11.9,10.9–13.1)	1818(43)	104(6)	2060(41)	359(17)
≥ 65	1182(13)	189(16.0,13.8–18.4)	545(13)	50(9)	636(13)	139(22)
Marital status						
Alone	1049(11)	146(13.9,11.8–16.4)	101(2)	5(5)	948(19)	141(15)
Married	8210(89)	817(10.0,9.3–10.7)	4173(98)	199(5)	4037(81)	618(15)
Ethnicity						
Persian	5215(56)	627(12.0,11.1–13.0)	2367(55)	124(5)	2848(57)	503(18)
Non-Persian	4047(44)	337(8.3,7.5–9.3)	1908(45)	80(4)	2139(43)	257(12)
SES						
Low	2419(26)	209(8.6,7.5–9.9)	973(23)	50(5)	1446(29)	159(11)
Low-middle	2499(27)	265(10.6,9.4–12.0)	1094(26)	50(5)	1405(28)	215(15)
Middle-high	2046(22)	224(10.9,9.6–12.5)	1016(24)	43(4)	1029(21)	181(18)
High	2299(25)	266(11.6,10.2–13.0)	1192(28)	61(5)	1107(22)	205(19)
Education						
Illiterate	4538(49)	564(12.4,11.4–13.5)	1336(31)	73(5)	3202(64)	491(15)
Literate	4717(51)	400(8.5,7.7–9.4)	2935(69)	131(4)	1782(36)	269(15)
Physical activity						
Low	3060(33)	471(15.4,14.0–6.8)	1034(24)	95(9)	2026(41)	376(19)
Intermediate	3056(33)	322(10.5,9.4–11.8)	1173(27)	42(4)	1883(38)	280(15)
High	3146(34)	171(5.4,4.7–6.3)	2068(48)	67(3)	1078(22)	104(10)
BMI (kg/m^2^)						
< 25	4100(44)	290(7.1,6.3–7.9)	2442(57)	96(4)	1658(33)	194(12)
< 30 & > 25	3442(37)	386(11.2,10.1–12.4)	1428(33)	80(6)	2014(40)	306(15)
≥ 30	1727(19)	288(16.7,14.8–18.7)	407(10)	28(7)	1319(26)	260(20)
Waist-to-height ratio						
Normal	3936(42)	208(5.3,14.8–18.7)	2692(63)	92(3)	1244(25)	116(9)
At-risk	5333(58)	756(14.2,14.8–18.7)	1585(37)	112(7)	3747(75)	644(17)
Waist-to-hip ratio						
Normal	1842(20)	69(3.7,2.9–4.7)	1294(30)	30(2)	548(11)	39(7)
At-risk	7375(80)	891(12.1,11.3–12.9)	2962(69)	174(6)	4413(88)	717(16)
TG (mg/dL)						
< 150	5638(61)	504(8.9,8.2–9.8)	2660(62)	109(4)	2978 (60)	395 (13)
150–199	1678(18)	165(9.8,8.4–11.5)	738(17)	37(5)	940 (19)	128 (14)
200–499	1853(20)	283(15.3,13.5–17.2)	828(19)	57(7)	1025 (21)	226 (22)
≥ 500	100(1)	12(12,6.2–21.0)	51(1)	1(2)	48 (1)	11 (23)
LDL (mg/dL)						
< 100	4016(43)	477(11.9,10.8–13.0)	2072(48)	132(6)	1944(39)	345(18)
100–129	3140(34)	283(9.0,8.0–10.1)	1427(33)	46(3)	1713(34)	237(14)
≥ 130	2113(23)	204(9.7,8.4–11.1)	778(18)	26(3)	1334(27)	178(13)
HDL						
High risk	538(6)	59(11,9.1–13.0)	369(9)	23(6)	169(3)	36(21)
Low risk	8741(94)	905(10.4,9.5–13.9)	3908(91)	181(5)	4822(97)	724(15)
Alcohol ever used						
No	9066(98)	955(10.5,9.9–11.2)	4109(96)	200(5)	4957(99)	755(15)
Yes	196(2)	9(4.6,2.1–8.7)	166(4)	4(2)	30(1)	5(17)
Tobacco ever used						
No	5709(62)	489(8.6,7.8–9.4)	3038(71)	128(4)	2671(54)	361(14)
Yes	3537(38)	472(13.3,12.2–14.6)	1231(29)	75(6)	2306(46)	397(17)
Cigarette ever used						
No	7345(79)	865(11.8,11.0–12.6)	2402(56)	112(5)	4943(99)	753(15)
Yes	1917(21)	99(5.2,4.2–6.3)	1873(44)	92(5)	44(1)	7(16)
Current Cigarette user						
No	7953(86)	913(11.5,10.7–12.2)	3007(70)	162(5)	4946(99)	751(15)
Yes	1296(14)	50(3.9,2.9–5.1)	1262(30)	42(3)	34(1)	8(24)
Opiate ever used						
No	8488(92)	923(10.9,10.2–11.6)	3532(83)	167(5)	4956(99)	756(15)
Yes	774(8)	41(5.3,3.8–7.2)	743(17)	37(5)	31(1)	4(13)
Comorbidities						
No Chronic Disease	3273(35)	49(1.5,1.1–2.0)	2012(47)	7(0)	1261(25)	42(3)
One or two	4255(46)	369(8.7,7.8–9.6)	1784(42)	91(5)	2471(50)	278(11)
Multi-morbidity	1741(19)	546(31.4,28.8–34.1)	481(11)	106(22)	1259(25)	440(35)

BMI, body mass index; TG, triglyceride; LDL, Low density lipoprotein; HDL, high density lipoprotein.

## Medication Profile

 Drugs for the alimentary tract and metabolism disorders were the most common first-level ATC drugs consumed by the participants who were on polypharmacy, followed by the cardiovascular drugs class. The prevalence of consumption of various ATC first level drug classes is presented in Table 2.

**Table 2:** Prevalence of First-Level ATC Medication Use in Percent

**Class**	**Class Title **	Prevalence of Medication Use in Percent*							
		**Among All Participants**				**Among Participants on Polypharmacy**			
		**Total**	**Male**	**Female**	**Elderly**	**Total**	**Male**	**Female**	**Elderly**
A	Alimentary tract and metabolism	25.7	19.3	31.1	34.9	76.7	70.6	78.3	72.5
C	Cardiovascular system	21.9	15.6	27.3	40.5	74.6	83.8	72.1	91.0
B	Blood and blood forming organs	18.2	5.9	28.7	17.5	58.9	52.0	60.8	56.1
G	Genito-urinary system and sex hormones	32.1	1.3	58.5	16.2	57.5	7.8	70.8	27.5
N	Nervous system	11.1	8.4	13.4	11.3	39.6	38.2	40.0	38.1
M	Musculo-skeletal system	10.3	8.1	12.3	21.5	36.4	40.2	35.4	46.6
H	Systemic hormonal preparations**	3.9	1.9	5.7	3.1	12.9	7.4	14.3	11.6
R	Respiratory system	2.3	2.2	2.5	3.7	7.7	10.3	7.0	10.6
J	Anti-infectives for systemic use	1.9	2.2	1.6	2.3	5.5	9.3	4.5	2.1

*Drug classes with a frequency of more than 5.0% among participants on polypharmacy are shown. Drugs are sorted based on total prevalence among all participants on polypharmacy. ** Excluding sex hormones and insulin.

 Based on our results, 58.2% of all women, 36% of women older than 60 years, and 70.1% of women who were on polypharmacy were using contraceptives (G03A). These made sex hormones (G03) the most frequent second-level ATC drug class consumed by the participants. The prevalence of consumption of various ATC second level drug classes is presented in Table 3.

**Table 3:** Prevalence of Second-Level ATC Medication Use in Percent

**Class**	**Class Title **	Prevalence of Medication Use in Percent*							
		**Among All Participants**				**Among Participants on Polypharmacy**			
		**Total**	**Male**	**Female**	**Elderly**	**Total**	**Male**	**Female**	**Elderly**
G03	Sex hormones and modulators of the genital system	31.5	0.2	58.4	14.6	55.7	1.5	70.3	24.9
A02	Drugs for acid related disorders	18.5	14.6	21.8	26.7	54.3	52.5	54.7	58.7
C07	Beta blocking agents	10.4	6.3	13.9	18.2	45.1	48.5	44.2	55.6
C10	Lipid modifying agents	7.5	5.5	9.2	13.0	37.8	49.5	34.6	44.4
B03	Anti-anemic preparations	13.6	1.2	24.3	6.5	36.6	8.3	44.2	17.5
M01	Anti-inflammatory and anti-rheumatic products	9.9	7.8	11.7	21.1	34.2	37.3	33.4	43.9
B01	Antithrombotic agents	5.4	4.8	5.9	12.0	28.6	44.1	24.5	43.9
C01	Cardiac therapy	5.8	5.1	6.3	15.0	28.3	46.6	23.4	50.3
A10	Drugs used in diabetes	5.8	3.9	7.3	8.5	24.0	19.6	25.1	19.6
C08	Calcium channel blockers	5.1	3.1	6.8	11.7	23.4	21.6	23.9	37.0
N05	Psycholeptics	4.5	3.5	5.4	5.0	21.3	25.5	20.1	22.8
C09	Agents acting on the renin–angiotensin system	3.3	2.5	4.0	7.3	17.7	24.5	15.9	27.5
N06	Psychoanaleptics	2.5	1.5	3.4	2.6	13.4	12.7	13.6	11.6
A11	Vitamins	2.9	0.9	4.7	3.2	12.2	3.4	14.6	9.5
C03	Diuretics	1.5	1.4	1.5	3.2	9.0	17.2	6.8	14.3
N02	Analgesics	3.6	3.2	4.0	3.1	8.5	7.4	8.8	5.3
H03	thyroid therapy	2.8	1.0	4.4	2.4	7.4	1.0	9.1	7.9
N03	Anti-epileptic	1.3	1.1	1.5	1.4	6.2	5.9	6.3	5.8
N07	other nervous system drugs	1.6	0.8	2.2	1.0	5.6	3.4	6.2	2.6
H02	Corticosteroids for systemic use	1.1	0.9	1.3	0.8	5.5	6.4	5.3	3.7
J01	Antibacterials for systemic use	1.7	2.0	1.5	2.0	5.2	8.3	4.3	2.1

*Drug classes with a frequency of more than 5% among participants on polypharmacy are shown. Drugs are sorted based on total prevalence among all participants on polypharmacy.

**Figure 1:**
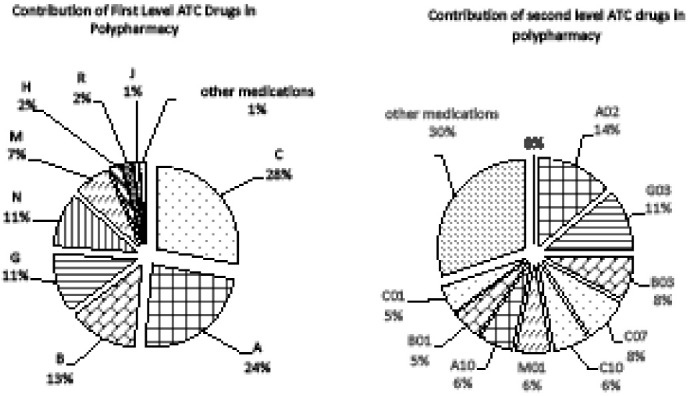
** Contribution of Medications to Polypharmacy;** the share of each drug class in all the medications consumed by the participants who were on polypharmacy is calculated. **C: **Cardiovascular system, **A:** Alimentary tract and metabolism, **B:** Blood and blood forming organs, **G:** Genito-urinary system and sex hormones, **N**: Nervous system, **M: **Musculo-skeletal system, **H:** Systemic hormonal preparations, excluding insulins and sex hormones, **R: **Respiratory system, **J**: Anti-infectives for systemic use. **A02: **Drugs for acid related disorders, **G03: **Sex hormones and modulators of the genital system, **B03:** Anti-anemic preparations, **C07: **Beta blocking agents, **C10:** Lipid modifying agents, **M01:** Anti-inflammatory and anti-rheumatic products, **A10:** Drugs used in diabetes, **B01: **Antithrombotic agents, **C01:** Cardiac therapy

 Cardiovascular drugs constituted about 28% of all medications consumed by the study participants who were on polypharmacy. Anti-acids (ATC A02), sex hormones (ATC G03), and anti-anemic preparations (ATC B03) accounted for more than 30% of all medications used in polypharmacy combined (Figure 1). Detailed contributions of medication classes to polypharmacy are presented in Supplementary File 1 (Tables S1 and S2). The difference between the numbers in Tables 2 and 3 (prevalence) with Figure 1 (contribution) is that the numbers in the tables are showing the prevalence of individuals consuming at least one drug of each drug class, but the numbers in Figure 1 show the share of each drug classes in all drugs consumed.

###  Correlates of Polypharmacy 

 Totally, 9230 of 9269 participants had no missing variables. After adjustment for potential confounders, there were significant associations between gender (APR = 1.89, 95% CI: 1.56 to 2.29), education (APR = 1.16, 95% CI: 1.0 to 1.34), and ethnicity (APR = 0.81, 95% CI: 0.72 to 0.91) with the prevalence of polypharmacy (Table 4).

**Table 4:** Correlates of the Prevalence of Polypharmacy among Participants of the PCS, Southwestern Iran

**Variable**	**Crude Prevalence Ratio (95% CI)**	**APR (95% CI)**
Gender		
Male	1 (ref)	1 (ref)
Female	3.19 (2.75–3.71)	1.89 (1.56–2.29)
Age group (y)		
40–49	1 (ref)	1 (ref)
50–64	1.61 (1.4–1.85)	1.37 (1.19–1.58)
≥ 65	2.16 (1.82–2.55)	1.5 (1.24–1.8)
Physical activity		
Low	1 (ref)	1 (ref)
Intermediate	0.68 (0.6–0.78)	0.79 (0.69–0.89)
High	0.35 (0.3–0.42)	0.63 (0.53–0.74)
Waist-to-height ratio		
Normal	1 (ref)	1 (ref)
At-risk	2.68 (2.31 - 3.11)	1.3 (1.11–1.53)
Waist-to-hip ratio		
Normal	1 (ref)	1 (ref)
At-risk	3.23 (2.54–4.1)	1.42 (1.1–1.83)
TG		
Normal	1 (ref)	
At-risk	1.27 (1.19–1.35)	
LDL		
Normal	1 (ref)	1 (ref)
At-risk	0.88 (0.81–0.95)	0.8 (0.74–0.86)
Marital status		
Alone	1 (ref)	1 (ref)
Married	0.71 (0.61–0.84)	1.26 (1.08–1.48)
Ethnicity		
Persian	1 (ref)	1 (ref)
non-Persian	0.69 (0.61–0.79)	0.81 (0.72–0.91)
SES		
Low	1 (ref)	1 (ref)
Low-middle	1.23 (1.03–1.46)	1.2 (1.02–1.41)
Middle-high	1.27 (1.06–1.52)	1.31 (1.1–1.55)
High	1.34 (1.13–1.59)	1.36 (1.14–1.61)
Education		
Illiterate	1 (ref)	1 (ref)
Literate	0.68 (0.6–0.77)	1.16 (1.0–1.34)
Tobacco ever used		
No	1.0 (ref)	1.0 (ref)
Yes	1.56 (1.38–1.76)	1.13 (1.01–1.26)
Cigarette ever used		
No	1 (ref)	1.0 (ref)
Yes	0.44 (0.36–0.54)	1.28 (0.96–1.7)
Cigarette now used		
No	1 (ref)	1 (ref)
Yes	0.34 (0.25–0.44)	0.63 (0.44–0.9)
Comorbidities		
No Chronic Disease	1 (ref)	1.0 (ref)
One or Two	5.79 (4.31–7.78)	4.61 (3.42–6.21)
Multi-morbidity	20.95 (15.73–27.9)	14.16 (10.52–19.05)

Ref, reference category; BMI, body mass index; TG, triglyceride; LDL, Low density lipoprotein; HDL, high density lipoprotein; APR, Adjusted prevalence ratio.

 Correlation of gender and education level with polypharmacy fades with excluding Contraceptives from the definition of polypharmacy. Also, by ignoring the cardiovascular drugs, the association of SES and age with polypharmacy becomes statistically insignificant (Supplementary File 1, Table S3).

## Discussion

 One-tenth of the general adult population, 5% of males, 15% of women, and 16% of older adults, were on polypharmacy. Multi-morbidity was the major correlate of polypharmacy with an APR of 14. Females with higher SES had a higher prevalence of polypharmacy. Sex hormones and modulators of the genital system (ATC G03), drugs for acid-related disorders (ATC A02), anti-anemic preparations (ATC B03), beta-blocking agents (ATC C07), anti-inflammatory and anti-rheumatic products (ATC M01) were the most prevalent medications among all the study participants.

###  Prevalence of Polypharmacy

 The prevalence of polypharmacy in our study was 10.4% which increased with age such that 16% of older adults (age ≥ 65 years) were on polypharmacy. To the best of our knowledge, no study has ever reported the prevalence of polypharmacy in middle-aged Iranians. Previous studies from urban regions of Iran reported that more than 20- 50% of Iranian older adults are exposed to polypharmacy.^23,24,25^ A study in more developed countries showed that the prevalence of polypharmacy in older adults of the general population in 18 developed countries (17 European and Israel) was between 26% and 40%.^26^ Accordingly, our study results highly suggested that the prevalence of polypharmacy in our setting at least in the elderly age group (age ≥ 65 years) could be lower than that in other regions. This could be a result of the low burden of inappropriate polypharmacy in our sample and thus desirable, but on the other hand, could be a result of medication underuse and the insufficiency of appropriate polypharmacy and thus alarming.

###  Medication Profile

 The most frequent medications consumed by the participants who were on polypharmacy were cardiovascular drugs (C), hormonal contraceptives (G03A), anti-acids (A02), anti-inflammatory (M01), and anti-anemic preparations (B03). Studies from several countries including those conducted in Iran have shown that cardiovascular drugs, gastrointestinal drugs, and analgesics are among the most prevalent medications used by all individuals, and also among patients exposed to polypharmacy.^8,27,28^ Although the prevalence of polypharmacy in the study setting was lower than other regions, hormonal contraceptives and anti-anemic preparations were used much more by our study participants than other regions, even in other regions of Iran.^8,27,28^ This could be a clue to the intense value of irrational use of these drug classes in our sample.

 About 58% of women older than 40 years were using oral or injectable hormonal contraceptives which is very high. This prevalence had been around 18% among Canadian females older than 40 years.^28^ A previous report from Iran also revealed lower rates of prevalence of hormonal contraceptive usage.^29^ This difference could be explained to an extent by reviewing family planning policies in Iran. Free contraceptives and ferrous sulfate pills had been available for every female without a physician prescription till 2014.^30^ Additionally, the extensive coverage of the Iranian primary healthcare network in rural regions, including the catchment area of the PCS, contributed to a greater accessibility to hormonal contraceptives. The impact of these drugs on polypharmacy is substantial. By removing them from the definition of polypharmacy, the statistically significant difference in the prevalence of polypharmacy between females and males would diminish.

 Further studies are recommended to evaluate other possible causes of contraceptives consumption in this region, such as the prevalence of gynecological diseases that warrant hormonal contraceptive use, and the lower social and gender development in the study catchment area that may make the females even more responsible for birth control and is in favor of the methods that need minimum cooperation by men. Although further studies are required to assess the appropriateness of hormonal contraceptive selection in this population, the high prevalence (36%) of these medications in participants aged beyond 60 years warrants an abnormal pattern of prescription or dispensing of these drugs.

 About one-quarter of all females and one-half of women on polypharmacy were using anti-anemic drugs regularly, and as mentioned above, access of the study population to iron pills is extremely easy in the study setting. Several reasons may be involved in this anomaly, such as low health literacy in this population, higher incidence of iron deficiency, the inadequacy of oral supplements for treating iron deficiency, and lack of proper monitoring of patients on treatment. Further research is required to recognize the major reasons that are involved in this area.

 Anti-inflammatory and anti-rheumatic products were used regularly by 11.7% of women and 7.8% of men; they were also the second most prevalent class of medicine in the elderly age group following acid-related drugs. Aspirin and acetaminophen are not included in this class. The point prevalence of Non-aspirin NSAID regular use in the USA was estimated at 11.6% from 1999–2004.^31^ This percentage was 9.1 in mid-aged adults in Switzerland from 2009 to 2012.^8^ Although the comparisons of these numbers are not informative, it seems that the consumption of these medications is not very different between our sample and the general populations of those countries, which suggests that the method of gathering drugs information in the PCS dataset was comparably capable of identifying the OTC medication use.

 Seventy-five percent of participants who were on polypharmacy used cardiovascular drugs but these medications contributed only to 28% of polypharmacy; on the other hand, hormonal contraceptives, acid suppressants, anti-inflammatory, and anti-anemic preparations altogether accounted for more than 45% (Figure 1 and Supplementary File 1, Tables S1 and S2). It is notable that compared to cardiovascular drugs, these four groups of medications should be often prescribed or recommend for a shorter period, have alternatives, and are also usually not necessary to use on a regular daily basis.^32,33^ Therefore, the irrational use of these four medication classes should be further studied for improving polypharmacy in similar regions.

###  Correlates of Polypharmacy

 Female participants had a higher prevalence of polypharmacy. This gender imbalance is consistent with the findings of other studies.^8,34^ In our study, the highest differences in the prevalence of drug use between men and women were seen for contraceptives, anti-anemic, and vitamins. By excluding contraceptives from the definition of polypharmacy variable, the effect of gender became insignificant (Supplementary File 1, Table S3) which shows that this gender imbalance may result from the use of hormonal contraceptives by females. Along with gender, the effects of education level and marital status also became insignificant by excluding contraceptives; however, the association of all biological health-related factors remained significant (Supplementary File 1, Table S3). Thus, modifying the use of contraceptives by literate married females is a suitable option to improve appropriate polypharmacy.

 Higher SES was associated with a higher prevalence of polypharmacy and this relationship was particularly significant in females. Although previous studies were inconsistent, no significant relationship was found after adjusting for other confounding variables in most of them.^35,36^ Higher SES groups tend to have more healthcare utilization which may increase medication use^37^ and consequently lead to a higher prevalence of polypharmacy. On the other hand, they might be in a better physical health status, which decreases their need for medications.^38^ In our study, by excluding cardiovascular drugs, the association between SES and polypharmacy became insignificant (Supplementary File 1, Table S3). This is in favor of less health-seeking behavior, less diagnosis, prescribing, or accessibility of cardiovascular drugs (which are often essential) for the population of lower SES classes. This alarming association and inequity in providing healthcare and particularly accessibility of cardiovascular medicine to the lower SES group were also shown elsewhere.^39^ It seems that in this region and probably other developing regions, we are facing a form of irrational drug use that presents itself by using more inappropriate medicine and less appropriate medicine by educated individuals and those who are from lower SES, respectively. The former may be partially attributed to the easy accessibility and also health literacy, and the latter may be mostly attributed to health inequity.

 The APR of polypharmacy increases with age. Two factors may contribute to this association. First, cardiovascular drugs are common and prescribed in clusters in older adults. The second factor is the increase in prescribed drugs for the elderly due to prescribing cascade.^40^ With the exclusion of cardiovascular drugs from polypharmacy definition, multivariable analyses show that the effect of age fades (Supplementary File 1, Table S3). Therefore, the first factor may be the more significant in our case, which suggests that using cardiovascular polypills may reduce the polypharmacy-related adverse outcomes.

## Limitations of the Study

 Our study is not without limitations. First, by not separating appropriate drugs from inappropriate drugs for each individual, we could not distinguish between appropriate and inappropriate polypharmacy. Nevertheless, clues were recognized in our study in favor of the high prevalence of inappropriate polypharmacy and low frequency of appropriate polypharmacy. Second, the duration of medication use was not documented. By analyzing those data, one may more precisely investigate the burden of each drug on the prevalence of polypharmacy, and differentiate between polypharmacy that results from chronic conditions and polypharmacy that results from acute diseases.

 In conclusion, the prevalence of polypharmacy in southwestern Iran is low, especially among males. Despite the low prevalence of polypharmacy, three clues are found in this study that may show the high burden of irrational use of medicine in this population. First, the use of anti-anemics is high in our sample; one-half of women who were on polypharmacy were taking these drugs. Second, the prevalence of hormonal contraceptives in women beyond 50 years of age is terrifyingly high. Third, the rule of cardiovascular drugs in polypharmacy is prominent solely in individuals with higher SES after adjustment for several biological, anthropometric and behavioral factors and this can be a sign of medicine underuse in participants with low SES. Modifying the accessibility of anti-anemic and hormonal contraceptives and cardiovascular drugs is recommended, but more research is required to adjust this modification.

## Supplementary Files

Supplementary file 1 contains Tables S1-S3.
