# Supplementary material for: Prevalence and Correlates of Polypharmacy, and Drug Utilization Pattern in a Semi-urban Population: Results from the Pars Cohort Study
Source: Arch Iran Med. 2023 Mar 1;26(3):156–65. doi: 10.34172/aim.2023.24 (PMC10685724; doi:10.34172/aim.2023.24)
Supplement: Supplementary file 1 — contains Tables S1-S3. [file aim-26-156-s001.pdf]

Arch Iran Med. March 2023;26(3):156-165

10.34172/aim.2023.24

http://journalaim.com

Supplementary file 1

Prevalence and Correlates of Polypharmacy, and Drug Utilization Pattern in a Semi-Urban Population: Results from the Pars Cohort Study

Table S1. Contribution of first level ATC drug class to polypharmacy

		Contribution in Polypharmacy			
		**All**	**Male**	**Female**	**Elderly**
C	Cardiovascular system	28.0	36.9	25.6	39.2
A	Alimentary tract and metabolism	23.5	22.0	23.9	22.5
B	Blood and blood forming organs	13.2	11.2	13.8	10.9
G	Genito-urinary system and sex hormones	11.5	1.5	14.1	4.6
N	Nervous system	10.5	12.7	9.9	8.4
M	Musculo-skeletal system	6.9	7.7	6.7	9.1
H	Systemic hormonal preparations, excluding sex hormones and insulin	2.1	1.4	2.3	1.8
R	Respiratory system	1.9	3.2	1.6	2.2
J	Anti-infectives for systemic use	1.0	2.1	0.8	0.3

Table S2. Contribution of first level ATC drug class to polypharmacy

		Contribution in Polypharmacy			
		**All**	**Male**	**Female**	**Elderly**
A02	Drugs for acid related disorders	13.9	14.2	13.8	14.8
G03	Sex hormones and modulators of the genital system	11.1	0.2	14.0	4.1
B03	Anti-anemic preparations	7.9	1.4	9.6	3.1
C07	Beta blocking agents	7.9	8.5	7.7	9.6
C10	Lipid modifying agents	6.3	8.3	5.8	7.2
M01	Anti-inflammatory and antirheumatic products	6.3	6.9	6.2	8.5
A10	Drugs used in diabetes	6.2	5.3	6.4	4.7
B01	Antithrombotic agents	5.3	9.8	4.2	7.8
C01	Cardiac therapy	5.0	8.8	4.0	8.6
N05	Psycholeptics	4.1	6.1	3.6	3.6
C08	Calcium channel blockers	3.8	3.5	3.9	5.9
C09	Agents acting on the renin–angiotensin system	2.8	3.9	2.5	4.3
N06	Psychoanaleptics	2.6	2.9	2.6	2.1
A11	Vitamins	2.1	0.5	2.6	1.6
C03	Diuretics	1.6	3.6	1.1	2.7
N02	Analgesics	1.5	1.4	1.5	1.0
H03	Thyroid therapy	1.2	0.2	1.5	1.2
N03	Anti-epileptic	1.1	1.2	1.1	1.0
H02	Corticosteroids for systemic use	0.9	1.2	0.9	0.6
J01	Antibacterials for systemic use	0.9	1.7	0.7	0.3
N07	other nervous system drugs	0.9	0.5	1.0	0.4

Table S3. Multivariable analysis for finding correlates of polypharmacy and by excluding specific medication classes

Variable	Including all Medications		Excluding contraceptive		Excluding Cardiovascular Drugs		Excluding A02 and A10	
	APR	*P*-Value	APR	*P*-Value	APR	*P*-Value	APR	*P*-Value
Gender								
Male	1(ref)				1(ref)		1(ref)	
Female	1.89	<0.001	NA	NA	3.58	<0.001	1.82	<0.001
Age group								
40–49	1(ref)		1(ref)				1(ref)	
50–64	1.37	<0.001	1.57	<0.001	NA	NA	1.43	<0.001
≥65	1.5	<0.001	1.8	<0.001	NA	NA	1.62	<0.001
Physical activity								
Low	1(ref)		1 (ref)		1(ref)		1(ref)	
Intermediate	0.79	<0.001	0.69	<0.001	0.8	0.014	0.8	0.006
High	0.63	<0.001	0.58	<0.001	0.6	<0.001	0.55	<0.001
Waist to height ratio								
<0.55	1(ref)		1(ref)				1(ref)	
≥0.55	1.3	0.001	1.29	0.007	NA	NA	1.69	<0.001
Waist to hip ratio								
Normal	1(ref)		1(ref)		1(ref)			
At risk	1.42	0.008	1.56	0.004	1.4	0.03	NA	NA
Marital status								
Alone	1(ref)				1(ref)		1(ref)	
Couple	1.26	0.003	NA	NA	1	0.003	1	0.009
Ethnicity								
Persian	1(ref)		1 (ref)		1 (ref)			
Non-Persian	0.81	<0.001	0.84	0.015	0.75	0.002	NA	NA
Socio-economic status								
Low	1(ref)		1 (ref)				1(ref)	
Low-middle	1.2	0.027	1.21	0.048	NA	NA	1.27	0.031
Middle-high	1.31	0.002	1.35	0.003	NA	NA	1.44	0.001
High	1.36	<0.001	1.39	0.001	NA	NA	1.59	<0.001
Education								
Illiterate	1(ref)				1(ref)			
Literate	1.16	0.043	NA	NA	1.38	<0.001	NA	NA
Tobacco ever used								
No	1(ref)		1 (ref)				1(ref)	
Yes	1.13	0.037	1.16	0.023	NA	NA	1.19	0.023
Cigarette ever used								
No	1(ref)							
Yes	1.28	0.092	NA	NA	NA	NA	NA	NA
Cigarette now used								
No	1(ref)		1(ref)					
Yes	0.63	0.01	0.76	0.052	NA	NA	NA	NA
multi-morbidity								
No dx	1(ref)		1(ref)		1(ref)		1(ref)	
1 or 2	4.61	<0.001	7.39	<0.001	3.31	<0.001	4.18	<0.001
Multimorbidity	14.16	<0.001	28.99	<0.001	10.62	<0.001	12.32	<0.001
TG								
Normal	1(ref)		1(ref)					
At risk	1.1	0.002	1.1	0.009	NA	NA	NA	NA
LDL								
Normal	1 (ref)		1 (ref)					
At risk	0.8	<0.001	0.79	<0.001	0.9	0.044	0.75	<0.001

NA, not applicable; TG, triglycerides; LDL, low density lipoproteins
